# Cynomolgus Monkeys With Spontaneous Type-2-Diabetes-Mellitus-Like Pathology Develop Alpha-Synuclein Alterations Reminiscent of Prodromal Parkinson’s Disease and Related Diseases

**DOI:** 10.3389/fnins.2020.00063

**Published:** 2020-02-07

**Authors:** Yan Sun, Chuang Guo, Lin Yuan, Wen Li, Zhan-You Wang, Feng Yue, Jia-Yi Li

**Affiliations:** ^1^Institute of Health Sciences, China Medical University, Shenyang, China; ^2^Institute of Neuroscience, College of Life and Health Sciences, Northeastern University, Liaoning, China; ^3^Neural Plasticity and Repair Unit, Wallenberg Neuroscience Center, Lund University, Lund, Sweden; ^4^Department of Neurobiology, Xuanwu Hospital of Capital Medical University, Beijing, China

**Keywords:** diabetes mellitus, Parkinson’s disease, α-synuclein, IAPP, protein aggregation, synucleinopathies

## Abstract

Available evidence suggests that diabetes mellitus (DM) is a non-genetic risk factor for Parkinson’s disease (PD). PD and DM have shared similarities in pathogenetic mechanisms, including age, environmental factors, inflammatory reaction, and protein aggregation, etc. α-Synuclein is the primary protein component in the protein inclusions in PD, while islet amyloid polypeptide (IAPP) aggregates to form amyloid structures in β cells in type 2 diabetes mellitus (T2DM). Pancreatic and cerebral functions, pancreas and brain α-synuclein deposition as well as striatal alterations, were assessed in spontaneously developed T2DM monkeys and age-matched normal monkeys. We demonstrated increased accumulation, aggregation, and phosphorylation of α-synuclein, and IAPP in the pancreatic islets of spontaneously developed T2DM monkeys, compared to the age-matched normal subjects. Double immunofluorescence analyses showed complete overlap between α-synuclein and IAPP in the pancreatic islets. In addition, in T2DM monkeys’ brain, we observed concomitantly increased accumulation and phosphorylation of α-synuclein in the cortex, pre-commissural putamen and dopaminergic neurons in the substantia nigra, which interestingly showed high correlation with levels of fasting plasma glucose (FPG), triglyceride (TG), and high density lipoprotein (HDL). Our data indicates the close association between IAPP and α-synuclein and the potential link between T2DM and PD, which implies that T2DM may facilitate PD disease onset and progress by interfering with the pathological protein aggregation both in the pancreatic islets and the brain.

## Introduction

Parkinson’s disease (PD) is the second most common neurodegenerative disease in human, after Alzheimer’s disease (AD) (Poewe et al., 2017). The main symptoms of PD are resting tremor, bradykinesia, postural instability, and rigidity, accompanied by a number of non-motor symptoms, such as sleep disorders, olfactory dysfunction, depression, constipation, etc. (Dickson et al., 2009). The pathological features of PD are the gradual loss of dopaminergic (DA) neurons in the substantia nigra pars compacta (SNpc), and the formation of eosinophilic inclusions, called Lewy Bodies (LBs) and Lewy neurites (LNs) (Fahn, 2003). The main protein component of LBs and LNs is α-synuclein, which selectively accumulates and aggregates in neurons and glial cells in PD and related diseases, such as dementia with Lewy body (DLB) and multiple system atrophy (MSA) (Kim et al., 2014). PD is a multifactorial disease, which may be attributed by a combined effect of different factors, such as genetic susceptibility, environmental toxins, and aging, etc.

Diabetes Mellitus (DM) is a systemic metabolic disease characterized by chronic progressive hyperglycemia, which is associated with abnormal insulin secretion and responses (American Diabetes Association, 2009), DM comprises Type 1 Diabetes Mellitus (T1DM) with insufficient insulin resulting from the destruction of pancreatic β cells, and Type 2 Diabetes Mellitus (T2DM) with an insulin relative deficiency, namely insulin resistance, which gives rise to a chronically elevated blood glucose level (American Diabetes Association, 2010). It has been well-documented that the main pathological features of T2DM are chronic insulin resistance, continuous β cell injury and β cell loss (Cerf, 2013). At present, T2DM, which contributes up to 90% of the DM population, has become one of the most common diseases affecting public health all over the world. Moreover, the incidence rate of T2DM increases rapidly in modern society. According to epidemiological estimation, by 2030, the number of people affected by the disease may reach approximately 366 million worldwide (Olokoba et al., 2012).

Emerging evidence has shown that PD is associated with T2DM. Epidemiological investigation shows that patients with T2DM have a higher risk (up to 38%) of suffering from PD than that of the normal population (Yue X. et al., 2016). In addition, earlier onset of DM is associated with an increased risk of suffering from PD (Wu et al., 2014). Moreover, T2DM, the world’s fastest-growing public health problem, can trigger a variety of complications in the central nervous system, including neurodegenerative diseases such as diabetic neuropathy and AD (Tabish, 2007; Boles et al., 2017). Clinical symptoms of DM such as hyperglycemia, high blood pressure, and high blood lipids can lead to systemic damage, including microcirculatory disorders, increased blood viscosity and impaired cell membrane function, which may lead to damage of the DA neurons in the midbrain (Cho et al., 2008; Cheung and Li, 2012; Afonso-Oramas et al., 2014). Patients with T2DM also exhibit motor symptoms such as abnormal postures and gait disorders (Alam et al., 2017). Conversely, approximately 50% of patients with PD have symptoms of abnormal glucose tolerance (Aviles-Olmos et al., 2013), among which PD patients with cognitive dysfunction are two times more likely to develop insulin resistance than patients without cognitive impairments (Munshi et al., 2006). However, to date, the molecular mechanisms underlying the interplay between T2DM and PD are still unclear.

α-Synuclein accumulation and aggregation play an important role in the pathogenetic process of PD (Bridi and Hirth, 2018). Studies have shown that many non-motor symptoms of PD, such as constipation, autonomic dysfunction, etc., may be related to the pathological deposition of α-synuclein in the peripheral nervous system (PNS) (Visanji et al., 2013; Borghammer et al., 2016; Borghammer, 2018). For example, the protein accumulates and phosphorylates in the enteric neurons of the gastrointestinal tract of mice in an age-dependent manner (Zhong et al., 2017; Chen et al., 2018). Different forms (monomers, oligomers or fibrils) of exogenous α-synuclein can be taken up by the nerve terminals in the intestinal tract and rapidly transmitted to the brain via the vagal nerve, after being injected into the gastrointestinal wall (Ulusoy et al., 2017; Kim et al., 2019). Additionally, α-synuclein is also found in the pancreatic β cells (Emamzadeh, 2016). The role of α-synuclein in the islet β cells is unclear, however, it appears that α-synuclein is present in the insulin secretory granules of islet β cells and can inhibit insulin secretion by binding to K_ATP_ channels (Geng et al., 2011), resulting in insulin reduction and aggravating the process of DM. Recently, the involvement of islet amyloid polypeptide (IAPP) as an amyloid-like protein in the pathogenesis of T2DM has drawn a significant amount of attention (Akter et al., 2016). IAPP is a polypeptide, which is composed of 37 amino acids, with a molecular weight of 3.85 kD (Stridsberg and Wilander, 1991). Under physiological conditions, it is stored in the secretory granules together with insulin and is secreted by islet β cells together with insulin at a ratio of 1:100, under the stimulation of physiological glucose and other chemical signals (Hou et al., 2009). IAPP has a series of physiological functions, including inhibiting the insulin-dependent cells on glucose utilization; reducing the emptying speed of the stomach and delaying the absorption of food by inhibiting the feeding center; inhibiting the secretion of insulin by blocking the acetylcholine nervous system; and inhibiting the secretion of glucagon from the islets to regulate blood sugar (Akter et al., 2016). IAPP in human and non-human primates has a very strong tendency to aggregate (Mukherjee et al., 2017). Under physiological conditions, IAPP and insulin are secreted together from the islet β-cell secretory vesicles with the stimulation of glucose and other nutrients, but the secretion of IAPP is much higher than insulin under the stimulation of high level glucose (Ahren and Gutniak, 1997). The increase of local IAPP concentration is the premise of IAPP fibrosis and aggregation in the islets (Abedini et al., 2018).

Although many studies regarding amyloid protein formation focus on disease-specific peptides individually, interactions among different disease-associated amyloid proteins may reflect the amyloidogenic pathways and pathogenicity of the structures physiologically unrelated to diseases. For example, IAPP can cross-react with prion protein (PrP) and β-amyloid fibers both *in vitro* and *in vivo* and form different compositions of amyloid proteins (Götz et al., 2013; Pillay and Govender, 2013). It has been shown that IAPP can accelerate α-synuclein amyloid formation *in vitro* (Horvath and Wittung-Stafshede, 2016) and the interaction between IAPP and α-synuclein in the pancreatic β cells of the patients with synucleinopathies has been detected (Martinez-Valbuena et al., 2018).

Here, we reported concomitant alterations of α-synuclein accumulation, aggregation, and phosphorylation in the pancreatic islets and different brain regions in aged (about 20 years old) cynomolgus monkeys with spontaneous T2DM, compared to the age-matched normal subjects. Double immunofluorescence labeling showed increased α-synuclein accumulation in dopaminergic neurons in the T2DM monkey’s brain. α-Synuclein was shown highly colocalized with IAPP in the T2DM monkey pancreas. Finally, we observed the close correlation between the levels of the biochemical indicators such as FPG, cholesterol (CHOL), high density lipoprotein (HDL), low density lipoprotein (LDL) and TG and α-synuclein level and phosphorylation, indicating that T2DM is a risk factor of PD, particularly inducing prodromal pathological alterations in PD and related diseases.

## Materials and Methods

### Animals

All animals in this study were randomizedly screened from the primate colony of a commercial primate farmer (Grandforest Primate Breeding Co. Ltd., Guangxi, China), in which the animals were group housed outdoors in 15 m × 24 m open area around with shelter room, there are feeding stations for animals to access the food and supplements, and space for animals to freely move or exercise. The animals were twice daily fed with grain based pellets diet, average 150–200 g per day for one monkey along with water available *ad libitum*, which contained protein (18.8%, W/W), fat (4.9%), CHOL (<0.0026%), and fiber(8.5%) with the remaining amount as carbohydrates (53.5%) and minerals. Additionally, animals were supplemented once daily with fresh fruits and vegetables. Seven aged cynomolgus monkeys (16–20 years old), diagnosed by elevated fast blood glucose level, and six age-matched normal monkeys were used in the project. All T2DM animals were naïve and naturally occurred, and never exposed from any interventions, including STZ-like intoxication, and the etiology of the T2DM monkeys remained to be unclear. The detailed screening protocol for T2DM can refer to the published studies (Yue F. et al., 2016; Yue et al., 2017). The cynomolgus monkeys were raised in the Guangxi Grandforest Primate Breeding Center. The experimental animals have detailed birth records and quarantine certificates.

### Biochemical Analyses

A set of biochemical and lipid markers, such as FPG, TG, CHOL, HDL, LDL and insulin in the blood were examined in the fasting condition in the morning; the procedures had been described previously (Yue F. et al., 2016). The monkey information and the levels of these markers are summarized in Table 1.

**TABLE 1:** Animals information and Biochemical Indicators level.

**ID**	**Groups**	**Birth Date**	**Age**	**Sex**	**FPG (mmol/L)**	**TG (mmol/L)**	**CHOL (mmol/L)**	**HDL (mmol/L)**	**LDL (mmol/L)**	**Insulin (mU/L)**
982311	T2DM	1998/3/1	16	♀	13.01	0.31	1.83	1.08	0.688	89.765
940790	T2DM	1994/1/1	20	♀	24.34	7.76	4.5	0.22	2.728	7.774
910136	T2DM	1991/1/1	23	♀	12.74	2.18	3.01	0.32	2.254	6.301
942055	T2DM	1994/3/20	20	♂	22.38	12.58	4.54	0.24	1.784	10.258
950145	T2DM	1995/1/5	19	♂	28.35	7.66	3.04	0.21	1.298	0.394
950135	T2DM	1995/1/1	20	♂	12.12	3.77	3.37	0.33	2.286	3.232
952044	T2DM	1995/2/4	20	♀	19.69	3.62	2.8	0.19	1.886	110.239
942303	Normal	1994/3/8	20	♀	4.44	0.43	3.11	1.36	1.664	7.821
962307	Normal	1995/10/14	19	♀	4.26	0.59	2.67	1.65	0.902	4.147
962305	Normal	1996/5/12	18	♀	5.69	0.46	2.04	1.17	0.778	12.879
932299	Normal	1993/11/8	21	♀	5.7	0.79	3.67	1.5	2.012	20.157
930840	Normal	1993/1/1	21	♀	2.83	1.14	3.87	1.97	1.672	11.701

### Sample Collection and Processing

All the animals were euthanized by administering the ketamine and then an overdose of sodium pentobarbital (>50 mg/kg), the transcardially perfused with ice-cold saline, followed with 4% paraformaldehyde (PFA). The brain was taken out and was post-fixed with the same fixative, for two overnights before rinsing in 0.1M PBS with 30% sucrose. Different peripheral tissues, including the pancreas, small intestine, and colon, etc. were also dissected and post-fixed in the same manner as the brain.

### Immunohistochemistry

The brains were coronally cut into 40 μm thick sections with a microtome (Leica, Germany). The sections were placed in an anti-freeze medium and stored in a −20°C freezer, until use. The pancreas was sliced into three portions, i.e., the pancreatic head, body, and tail, and was embedded into paraffin. The paraffin-embedded pancreatic head was cut into 5 μm thick sections and mounted on gelatin-coated glass-slides for immune-labeling.

Antigen retrieval of free-floating sections was achieved by incubating them in a citric acid buffer at 60°C for 30 min, and antigen retrieval of paraffin sections was performed by incubating them in the citric acid buffer for 30 min in a microwave oven at 80°C. Sections were quenched with 3% H_2_O_2_ in 10% methanol for 10 min before blocking (5% normal horse or goat serum in 0.1% BSA and 0.3% TritonX-100 in 0.01M PBS) for 1h. Sections were incubated with primary antibodies (diluted in 0.1% BSA and 0.3%TritonX-100 in 0.01M PBS) overnight at 4°C. The antibodies used are listed in Supplementary Table S1. After rinsing, biotinylated secondary antibodies (horse anti-mouse, goat anti-rabbit; dilution 1:400, Vector Laboratories) were applied for 1 h at room temperature, washed with PBS and followed by incubation with ABC complex (Vector Laboratories) for 1 h, and then incubation with DAB (Vector Laboratories) for the section coloration.

Paraffin sections were deparaffinized with xylene and then sequential decreasing concentrations of ethanol before use in the immunohistochemistry procedure as described above. After immunostaining, the sections were incubated with hematoxylin (Beyotime, C0107) for 5 min, rinsed for 5 min with water, and then differentiated with acid alcohol for 10 s.

### Immunofluorescence

For immunofluorescence double labeling of the paraffin-embedded sections, after dewaxing and antigen retrieval as described above, the sections were incubated with a mixture of primary antibodies overnight at 4°C, and then followed by incubation with Alexa Fluor 488 and Cy3-conjugated secondary antibodies for 2 h at room temperature, washed again with PBS, and incubated with 2-(4-Amidinophenyl)-6-indolecarbamidine dihydrochloride (DAPI, Beyotime, C1005) for 5 min, before covering with an antifade mounting medium (Beyotime, P0126). The images were acquired using a confocal laser scanning microscope (SP8, Leica, Germany).

### Quantitative and Statistical Analyses

In order to quantify the intensity of immunoreactivity after stained with a specific antibody in the cortex at the precommissural level (e.g., Figures 6–8), we took highly magnified images (with a 20x objective) from layer I to layer VI in a Nikon upward microscope (Nikon CI-CTLA, Japan). We defined the first picture from the superficial layer of the cortex as the layer I and II, the second picture as layer III, the third picture as layer VI and V, and the fourth picture as layer VI, respectively. We quantified the average immunoreactive intensity of different cortical layers, which were shown in Figures 6D, 7D, 8C. The average immunoreactive intensity of all the six cortical layers was used to perform the correlations with clinical biochemical indicators. The results were expressed as the mean ± standard error of the mean (SEM). The data were evaluated statistically by Independent-Samples *t*-test and bivariate correlations. The Pearson correlation coefficient (PCC), also referred to as Pearson’s r or the bivariate correlation, is a measure of the linear correlation between two variables X and Y. Here, we defined the biochemical indicators as X variables, correspondent proteins levels defined as Y variables. All results were analyzed using GraphPad Prism 7.0 software and IBM SPSS statistics 22 and differences were deemed to be statistically significant if *p* < 0.05.

To the quantification of dopaminergic neurons in the SNpc, due to the limited numbers of available sections containing the SNpc in each monkey, we have not been able to perform a systemic quantification, such as stereological analysis, to the dopaminergic neurons. Instead, we selected similar sections of the SNpc from T2DM and normal monkeys, numbers of TH-positive neurons were quantified and compared between T2DM and normal conditions. Student *t*-test was used to analyze the significance of the alterations.

## Results

### Biochemical Characterization of Diabetic Monkeys

**FIGURE 1:**
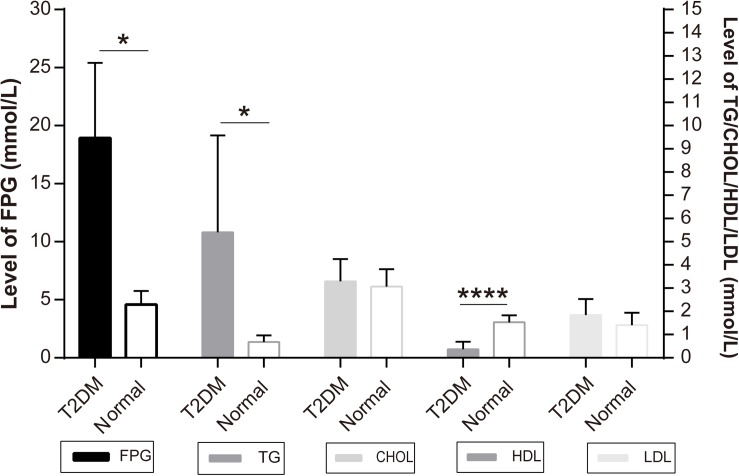
Independent samples test of diabetic and normal monkeys’ biochemical indicators: the *Y*-axis of FPG level is on the left and the *Y*-axis of TG, CHOL, HDL, LDL is on the right: *p < 0.05, ****p < 0.0001.

The monkeys with ages ranging from 16 to 23 years old were first screened for the level of FPG as the major diagnostic criteria of T2DM. According to the Standards of Medical Care in Diabetes -2019 (Diabetes Care 2019) to human subjects (American Diabetes Association, 2019), a level of FPG, ≥7.0 mmol/L, was diagnosed as diabetic (Table 1). All the diabetic monkeys exhibited elevated FPG, ranging from 12.12 to 28.35 mmol/L (male monkeys *n* = 3, female monkeys *n* = 4), the severity of elevated FPG level in the diabetic monkeys was not correlated to sex or age (Supplementary Tables S2, S3). Therefore, we pooled the male and female monkeys together in the different analyses below. In addition, we also examined alterations in dyslipidemia, particularly, for the alterations in total CHOL, TG, HDL, and LDL (Table 1). We observed a significant increase of TG (*p* < 0.05), but a decrease of HDL (*p* < 0.001) in diabetic monkeys compared to the age-matched control subjects (Figure 1). These data indicated that the monkeys used in the present study exhibited features reminiscent of T2DM.

### T2DM Induces Accumulation and Phosphorylation of α-Synuclein in Pancreatic Islets

**FIGURE 2:**
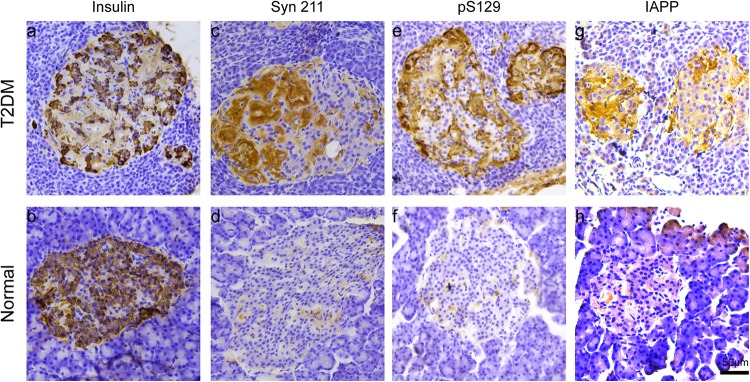
Microscopic images of insulin, α-synuclein, phosphorylated α-synuclein, and IAPP in the pancreatic islets. Immunohistochemical stainings with antibodies specifically against insulin **(a,b)**, α-synuclein (Syn 211) **(c,d)**, phosphorylated α-synuclein (pS129) **(e,f)**, and IAPP **(g,h)** in a 20-year-old female T2DM monkey (ID: DM952044) **(a,c,e,g)** and in a 21-year-old normal female monkey (ID: N930840) **(b,d,f,h)**. Scale bar = 50 μm.

We examined the presence of insulin in β cells in the pancreatic islets, using immunohistochemistry. Robustly, we observed significantly decreased insulin-positive cells in the pancreatic islets of all the biochemically diagnosed monkeys with diabetes compared to the normal subjects (Figures 2a,b), confirming the pathologic diagnosis of T2DM.

**FIGURE 3:**
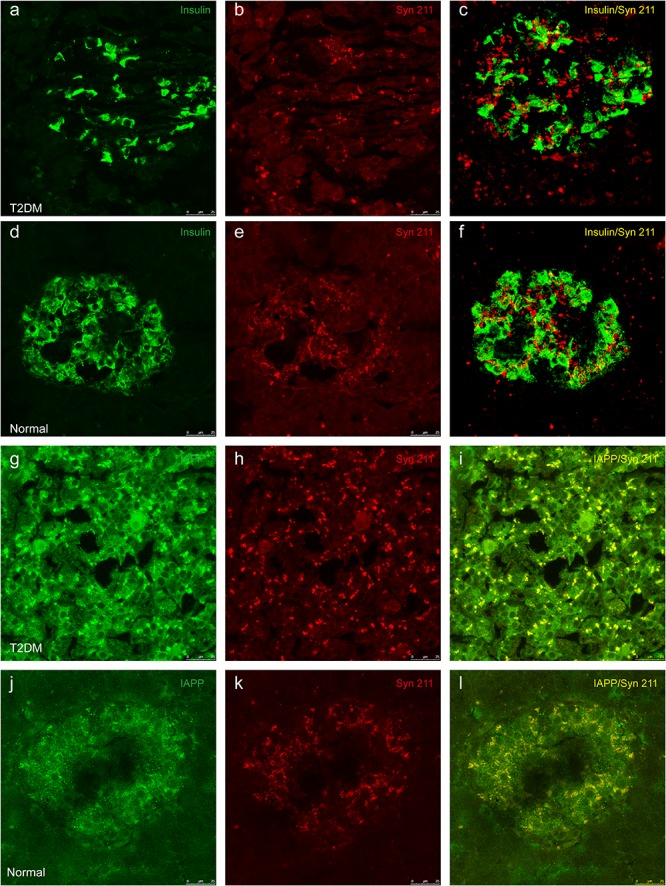
Double immunofluorescence images showing the presence of α-synuclein in insulin-positive cells **(a–f)** and colocalization between α-synuclein and IAPP **(g–l)**. Dual immunofluorescence labeled for insulin **(a,d)** and α-synuclein (Syn 211) **(b,e)** exhibits partial overlapping of these two proteins in pancreatic β cells from a 20-year-old female diabetic monkey (ID: DM952044) **(a–c)** and from a 21-year-old normal female monkey (ID: N930840) **(d–f)**. IAPP **(g,j)** and α-synuclein **(h,k)** are highly co-localized to each other in a 20-year-old T2DM male (ID: DM942055) **(g–i)** and a 20-year-old normal female (ID: N942303) **(j–l)**. Scale bar = 25 μm.

Previous evidence showed that low amounts of α-synuclein are present in pancreatic β cells at physiological conditions (Emamzadeh, 2016). α-Synuclein is localized in the insulin secretory granules of pancreatic β cells and can inhibit insulin secretion (Geng et al., 2011). In order to study whether more α-synuclein accumulates in the pancreatic islet cells in T2DM monkeys, we performed immunohistochemical analyses with different α-synuclein antibodies. Firstly, we detected high levels of cytoplasmic α-synuclein in pancreatic β cells in the pancreatic islets of all the T2DM monkeys compared with the age-matched normal subjects, using a human α-synuclein antibody (Syn 211), which recognizes normal and aggregated forms of α-synuclein (Figures 2c,d). In addition, stained with an antibody specifically against serine 129 phosphorylated-α-synuclein (pS129), we found that the phosphorylated form of the protein was significantly increased in the T2DM monkeys (Figures 2E,F). These results indicated that α-synuclein increased and phosphorylated in the pancreatic islets of T2DM monkeys. Double immunofluorescence with antibodies against α-synuclein and insulin showed partial co-localization between the two (Figures 3a–f and Supplementary Figure S1), implying that α-synuclein may not only be present in the pancreatic β cells but also in other cell types of the pancreas, such as α cells and δ cells.

### T2DM Triggers IAPP Accumulation, Which Was Closely Associated With α-Synuclein in Pancreatic Islets and Brain

**FIGURE 4:**
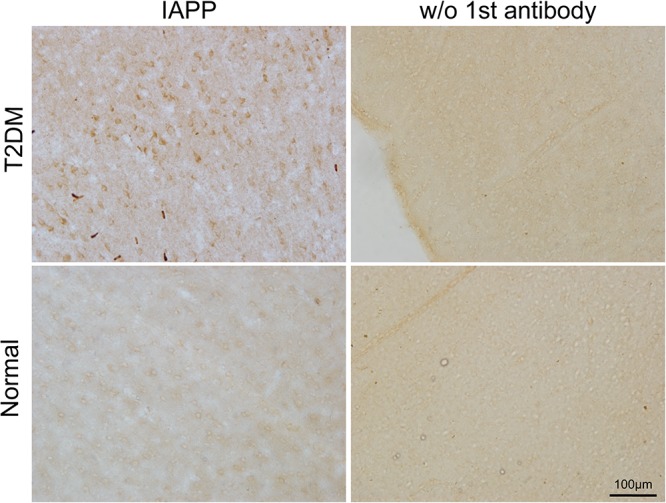
IAPP in the motor cortex of a diabetic and a normal monkey. Immunohistochemistry for IAPP in the motor cortex and corresponding negative control (**right panel**, incubation without the IAPP primary antibody) from a T2DM monkey **(upper panels)** (ID:DM940790) and an age-matched normal monkey **(lower panel)** (ID:N942303). Scale bar = 100 μm.

**FIGURE 5:**
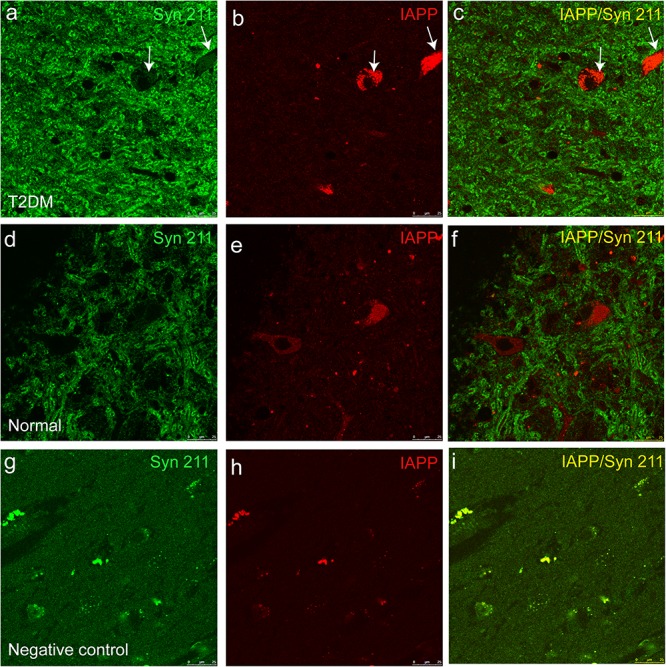
Dual immunofluorescence images for IAPP and α-synuclein (Syn 211) in the substantia nigra. The α-synuclein antibody **(a,d)** and an IAPP antibody **(b,e)** were used to stain the sections of the substantia nigra in a diabetic monkey (**a–c**, ID:DM940790) and a normal monkey **(d–f)**, ID:N942303) and a negative control (i.e., incubation without the primary antibodies) from the diabetic monkey (**g–i**, ID:DM940790). Arrows pointed the dopaminergic neurons containing detectable amounts of α-synuclein and IAPP. The fluorescent profiles in **(g–i)** are non-specifically stained structures containing autofluorescence. Scale bar = 25 μm.

IAPP is normally present in the pancreatic β cells and is highly associated with diabetic pathogenesis (Aston-Mourney et al., 2013; Abedini et al., 2018). Similar to the pattern of changes with α-synuclein, we detected an increased amount of IAPP in the pancreatic tissues of the T2DM monkeys compared to the normal ones (Figures 2g,h). Double immunofluorescence labeling with antibodies specifically against α-synuclein and IAPP revealed a complete co-localization between the two proteins. All of the α-synuclein positive profiles were overlapped with IAPP positive cells (Figure 3g–l). Interestingly, we also observed increased levels of IAPP in the cortical neurons (Figure 4) and dopaminergic neurons (Figure 5) of diabetic monkeys compared to the age-matched controls, the two proteins are partially colocalized with the dopaminergic neurons in the diabetic monkeys (Figures 5a–c, arrows), indicating the potential interplay of IAPP and α-synuclein in these neurons. These results indicated that α-synuclein and IAPP are closely associated with each other in the processes of their accumulation and aggregation in the pancreatic islets and also in the brain.

### Concomitant Accumulation and Phosphorylation of α-Synuclein in the Brain of T2DM Monkeys

**FIGURE 6:**
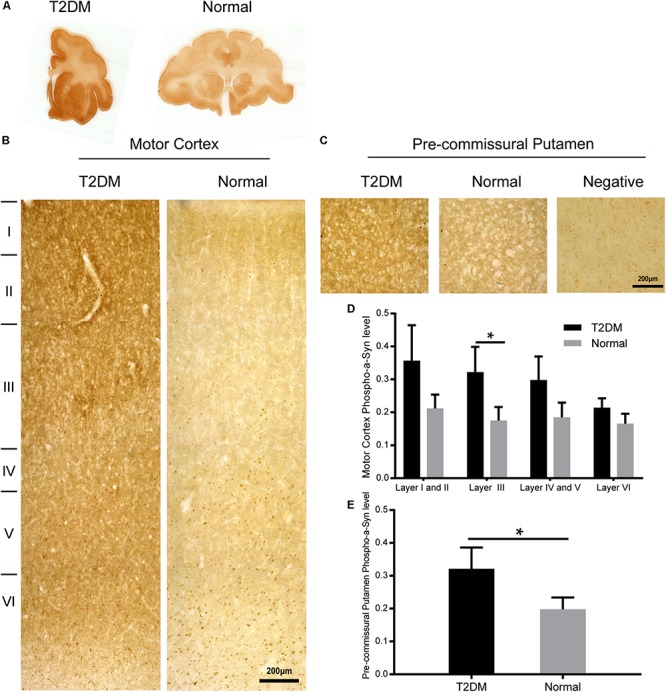
α-Synuclein in the motor cortex and the pre-commissural putamen of diabetic and normal monkeys. Immunohistochemistry for α-synuclein (Syn 211) in the motor cortex marked with estimated cortical layers **(B)** and in the pre-commissural putamen **(C)** of a T2DM monkey (left) and an age-matched normal monkeys (right). Macro-scanned images of α-synuclein stained brain sections **(A)**. A negative control image without the primary antibody incubation was also shown (right, in **C**). High power images were also shown in Supplementary Figure S2. Scale bars = 200 μm in **(B,C)**. Quantitative analysis of immunohistochemical intensity of α-synuclein in the motor cortex **(D)** and in the pre-commissural putamen **(E)**: Data is represented with the mean ± SE (*n* = 5∼7), *p < 0.05.

**FIGURE 7:**
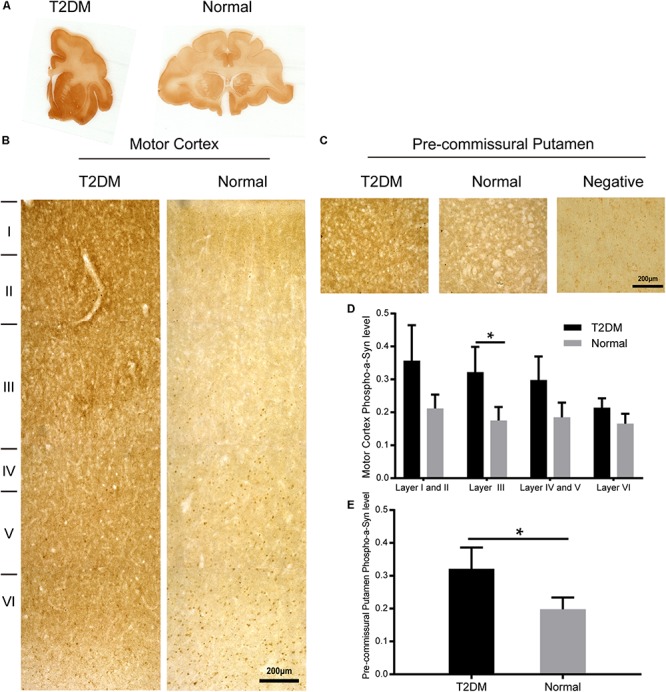
Phosphorylated α-synuclein in the motor cortex and the pre-commissural putamen of diabetic and normal monkeys. Immunohistochemistry for phosphorylated pS129 α-synuclein in the motor cortex marked with estimated cortical layers **(B)** and in the pre-commissural putamen **(C)** of a T2DM monkey (left) and an age-matched normal monkey (right). Macro-scanned images of the immunohistochemically stained sections **(A)**. A negative control image without the primary antibody incubation was also shown (right, in **C**). High power images were also shown in Supplementary Figure S3. Scale abrs = 200 μm in **(B,C)**. Quantitative analysis of immunohistochemical intensity of phosphorylated α-synuclein **(D,E)** in the motor cortex **(D)** and in the pre-commissural putamen **(E)**. Data is represented with the mean ± SE (*n* = 5∼7), *p < 0.05.

To determine whether there exist α-synuclein aggregation and/or α-synuclein pathological changes in the brains of the diabetic monkeys, we evaluated the presence and distribution of α-synuclein (Figure 6A), phosphorylated-α-synuclein (pS129) (Figure 7A) in the selected brain regions. Although we did not observe typical LBs nor LNs in either the diabetic or normal monkeys, the overall amount of α-synuclein increased in the motor cortex (Figure 6B) and the pre-commissural putamen, including the caudate nucleus and pre-commissural putamen (Figure 6C) under the diabetic conditions. Quantitative analyses of α-synuclein in the cortical regions showed that the amounts of α-synuclein accumulation appeared more robustly in the deeper cortical layers. α-Synuclein in the superficial layers (I-III) of the cortex appeared similar in intensity between the diabetic and normal monkeys, while the amounts of the protein were significantly higher in the deep layers (IV-VI) (Figures 6B,D and Supplementary Figure S2). In addition, more abundant α-synuclein appeared in the pre-commissural putamen of diabetic monkeys (Figures 6C,E and Supplementary Figure S2). Post-translational modifications, such as phosphorylation, commonly occur in pathological processes in PD and related disorders (Reynolds et al., 2007; Lashuel et al., 2013; Samuel et al., 2016). In the diabetic monkeys, phosphorylated α-synuclein (Figures 7A–E and Supplementary Figure S3) accumulated in different layers of the cortex and the pre-commissural putamen, indicating that persistent hyperglycemia may induce pathological posttranslational modifications of α-synuclein.

### Hyperglycemia Induces Synapse Loss and α-Synuclein Accumulation in Dopaminergic Neurons

**FIGURE 8:**
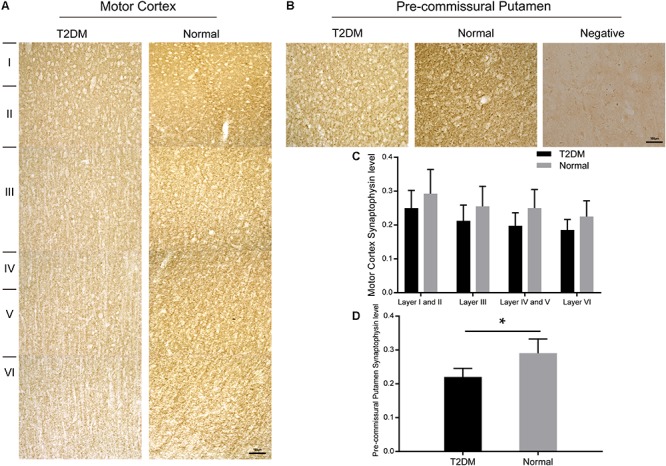
Immunohistochemically stained motor cortex **(A)** and pre-commissural putamen **(B)** with a synaptophysin antibody from a T2DM monkey (left) and an age-matched normal monkey (right). High power images were also shown in Supplementary Figure S4. Quantitative analyses of the immunohistochemical intensity of synaptophysin in the motor cortex **(C)** and in the pre-commissural putamen **(D)**. A negative control image without the primary antibody incubation was also shown (right, in **B**). Data was represented with the mean ± SE (*n* = 5∼7), *p < 0.05. Scale bars = 100 μm in **(A,B)**.

Synaptophysin is a presynaptic vesicle protein with four transmembrane domains. It is present abundantly in different types of synapses and is commonly used as a synaptic marker to detect the presence and density of synapse in (patho-)physiological conditions. Here, we detected a reduction trend in synaptophysin-positive puncta in different layers of the cortex of the diabetic monkeys. However, the reduction of synaptophysin immunoreativity became significant in the pre-commissural putamen of diabetic monkeys compared to that of the normal ones (Figures 8A–D and Supplementary Figure S4).

**FIGURE 9:**
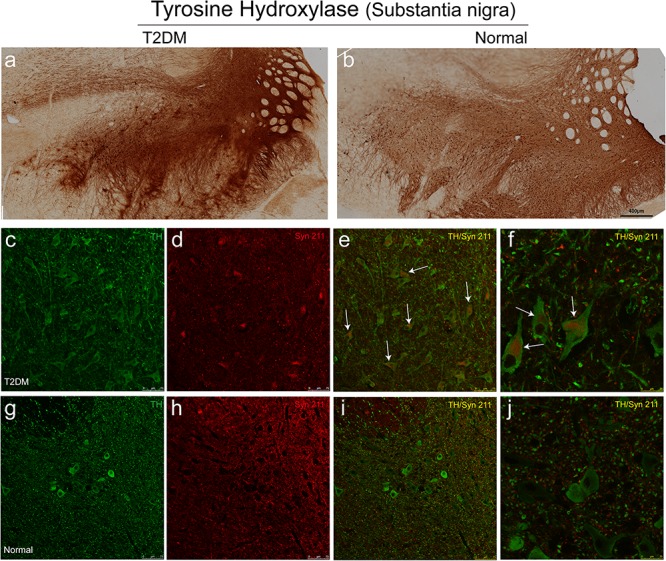
Immunohistochemical images showing presence of dopaminergic neurons of the substantia nigra in diabetic and normal monkeys. An antibody against tyrosine hydroxylase (TH) was used to stain the sections of the substantia nigra in diabetic **(a)** and normal **(b)** monkeys. Double immunofluorescence labeling with α-synuclein (Syn 211) (red in **d–f,h–j**) and TH (green in **c,e,f,g,i,j**) showed increased α-synuclein present in many TH-positive neurons in the diabetic monkey (arrows in **e,f**), compared to that in the age-matched normal monkey **(i,j)**. Scale bar = 400 μm in **(a,b)**; scale bar = 75 μm in **(c–e,g-i)**; scale bar = 25 μm in **(f,j)**. High power series images were also shown in the Supplementary Videos S1, S2.

Tyrosine hydroxylase (TH) is the rate-limiting, key enzyme for the synthesis of catecholamines, including dopamine, noradrenaline and adrenaline. We assessed whether hyperglycemia could cause TH positive dopaminergic degeneration. Using the immunohistochemical methods with a TH antibody, we did not observe a significant reduction of TH-positive neurons in the substantia nigra of T2DM monkeys (Figure 9a), compared to the normal subjects (Figure 9b). However, very interestingly, double immunofluorescence labeling with TH and α-synuclein antibodies showed that the majority of TH positive neurons (up to 90%) in the substantia nigra of the diabetic monkeys contained significantly increased levels of α-synuclein (Figures 9c–f, arrows, and Supplementary Video S1), compared to the ones in the normal monkeys (Figures 9g–j and Supplementary Video S2), implying that T2DM induces accumulation of α-synuclein in dopaminergic neurons, as an early feature of pathology (Li et al., 2008).

### Correlation Between Brain Pathological Development and the Levels of Plasma Glucose and Severity of Dyslipidemia

**FIGURE 10:**
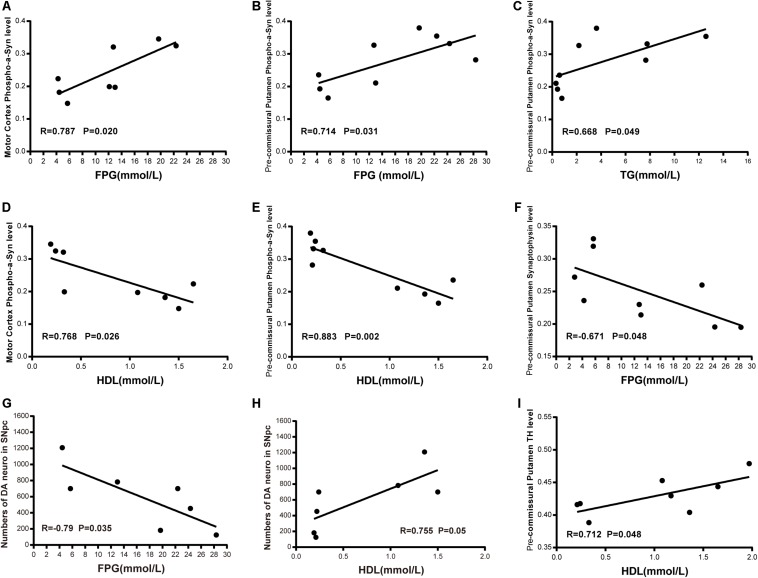
Correlation between biochemical indicators and phosphorylated-α-synuclein; synapse density in the motor cortex and the pre-commissural putamen; dopaminergic neurons and terminals in the substantia nigra and the pre-commissural putamen. Scatter diagram of Pearson correlation test between α-synuclein level or dopaminergic system and FPG level **(A,B,F,G)**, TG level **(C)**, HDL level **(D,E,H,I)**.

In order to assess potential correlations between the severity of T2DM and dyslipidemia, α-synuclein accumulation and phosphorylation in the brain, we performed co-efficient analyses between the related variables. We observed a significant positive correlation between the level of FPG and the robustness of phosphorylated α-synuclein (pS129) in the cortex and the pre-commissural putamen (Figures 10A,B). In addition, the level of TG also exhibited a positive correlation with phosphorylated α-synuclein in the pre-commissural putamen (Figure 10C). However, the level of HDL was negatively correlated to the levels of phosphorylated α-synuclein accumulation (Figures 10D,E), indicating that severity of T2DM is closely associated with the pathological development of PD and related diseases. Moreover, it appeared that synaptic density was negatively correlated to the level of FPG (Figure 10F). When assessing the severity of dopaminergic neuron loss in correlation to the levels of FPG and HDL, it was evident that more severe blood hyperglycemia-induced more abundant dopaminergic neuron loss (Figure 10G), while HDL levels showed a positive correlation to the number of dopaminergic neurons in the substantia nigra (Figure 10H) and nerve terminals in the pre-commissural putamen (Figure 10I).

## Discussion

Emerging epidemiological and clinical evidence suggests that PD and T2DM, the two age-related diseases, share certain common pathological mechanisms (Aviles-Olmos et al., 2013; Santiago and Potashkin, 2014). In the present study, we have demonstrated a close association of T2DM with PD and related diseases, such as DLB and MSA, all of which share similar neuropathology of α-synuclein aggregation in neurons or glia (Hasan and Mielke, 2019). From the neuropathological point of view, these diseases have also been called synucleinopathies (Goedert et al., 2017). Here, we studied the spontaneously developed T2DM cynomolgus monkeys and the age-matched normal subjects. We detected significantly increased α-synuclein and phosphorylated α-synuclein accumulation in the pancreatic β cells in the T2DM monkeys. We, for the first time, presented histological evidence of α-synuclein-IAPP association and co-localization in the pancreatic cells of non-human primate models. Concomitantly, we observed significantly increased accumulation and phosphorylation of α-synuclein in the motor cortex and the pre-commissural putamen as well as dopaminergic neurons in the substantia nigra of the diabetic monkeys, which is highly correlated to the levels of FPG, TG, and HDL.

### α-Synuclein Accumulation and Phosphorylation in Relation to the IAPP Aggregation in Diabetic Conditions

Despite some discrepancies, the majority of the previous reports suggested an association between diabetes and PD (D’Amelio et al., 2009; Palacios et al., 2011). T2DM patients exhibited an increased risk of developing PD (He et al., 2007; Hu et al., 2011; Wahlqvist et al., 2012), while PD patients displayed perturbed sugar metabolism (Konig et al., 2018). Some anti-diabetic drugs have also been proven to be beneficial for PD patients (Athauda et al., 2017). Moreover, impaired insulin signaling in PD, as corroborated by increased insulin plasma concentrations, implies the potential association between α-synuclein aggregation and insulin resistance (Bassil et al., 2017). Martinez-Valbuena et al. (2018) demonstrated the increased accumulation of phosphorylated α-synuclein and IAPP in the pancreatic β cells of the patients who suffered from different synucleinopathies, including PD, DLB, and incidental Lewy body disease. However, no correlative changes α-synuclein aggregation in the brain were described (Martinez-Valbuena et al., 2018). In the present study, we provided direct evidence of T2DM triggering α-synuclein accumulation, aggregation, and phosphorylation, not only in the pancreatic islets but also in various brain regions, including the cortical neurons and dopaminergic neurons in the substantia nigra. However, up to date, it is still not clear on the causal relationship between PD and T2DM.

T2DM, a chronic systematic metabolic disease, is characterized by pancreatic β cells dysfunction and insulin resistance, which are characterized by that insulin is not sensitive to glucose and subsequently develop hyperglycemia and pancreatic β cells loss (Hameed et al., 2015). It has been shown that IAPP plays a role in the development of T2DM. IAPP is co-secreted with insulin. The secretion level of IAPP is much higher than insulin under the stimulation of high glucose, the partial increase of IAPP is the precondition of IAPP fibrosis, aggregation, and deposition in islets. Several studies have implicated that IAPP aggregation can conversely aggravate the pathology of diabetes and accelerate the progression of diabetes (Mukherjee et al., 2015), but whether IAPP deposits is a cause or a consequence of T2DM is still unclear. Here, we detected the extensive IAPP aggregation in pancreatic β-cells, which was the primary pathological characteristic of T2DM. We also observed high co-localization of IAPP and α-synuclein. Emerging evidence suggests co-aggregation of different amyloid proteins. For example, Aβ appeared to accelerate the amyloid formation of IAPP *in vitro* (O’Nuallain et al., 2004) and IAPP was found as a protein component in Aβ plaques in AD transgenic mouse brains (Oskarsson et al., 2015). α-Synuclein can assemble via homologous and/or heterogenous oligomeric intermediates to amyloid fibrils under pathological conditions. It has been reported that both IAPP and pro-IAPP could promote and accelerate α-synuclein aggregation via directly interacting with each other *in vitro* (Horvath and Wittung-Stafshede, 2016). Our studies implied that IAPP may associate with the enhanced α-synuclein and phosphorylated α-synuclein accumulation and aggregation in pancreatic β cells and the brain of aged spontaneous T2DM non-human primate animals. Recently, Mukherjee et al. (2017) reported that pancreatic IAPP aggregates could seed endogenous IAPP and enhance IAPP misfolding and aggregations *in vitro* and *in vivo* models via prion-like manner. Similar α-synuclein propagation and aggregation have also been reported in the gastrointestinal tract, implicating gut-to-brain spread of α-synuclein (Holmqvist et al., 2014; Martinez-Valbuena et al., 2018; Kim et al., 2019). It is still not clear whether a heterologous seeding of amyloid forms of IAPP and α-synuclein between PD and T2DM may take place. Interestingly, we observed the IAPP accumulation in the cortex and the substantia nigra of diabetic monkeys. Furthermore, we observed partial colocalization between IAPP and α-synuclein. Further investigations will be needed on their interactions and (co-)aggregation in different models and different encephalic regions to reconcile this important issue. Moreover, dysfunction of lysosomal systems and the aggregation of α-synuclein into toxic fibrils, are thought to be critical steps in the process leading to dopaminergic neuron death in PD. Insulin signaling has been shown to influence lysosomal systems accompanied by increased expression of PD pathology (Matsuzaki et al., 2010; Willette et al., 2015). Available evidence has shown that insulin signaling can modulate the degradation of α-synuclein (Sharma et al., 2015). Therefore, it is conceivable that impaired insulin signaling in diabetic conditions may contribute to α-synuclein accumulation and aggregation in the pancreatic islets and also in the brain. Therefore, it is of interest to do more further works to verify how insulin signaling dysfunction play a role in amyloid protein accumulation in diabetic conditions.

### T2DM, a Prodromal Pathological Feature of PD?

In an epidemiological and retrospective nationwide cohort study from South Korea, individuals with the metabolic disease had a 24% higher risk of incident PD compared to the subjects without metabolic diseases (Nam et al., 2018), indicating that metabolic diseases, such as diabetes are risk factors for PD development. Our data here have provided the morphological and correlative evidence, confirming such an association between diabetes and PD. Very interestingly, we only observed a significant increase of α-synuclein and phosphorylated-α-synuclein in the pancreatic islets and the brain, accompanied with reduced synaptic profiles, but we did not find typical LBs and LNs in the diabetic monkeys. Increased α-synuclein accumulation occurred normally during the aging (Chu and Kordower, 2007). Compared to the age-matched normal monkeys, we reasoned that elevated α-synuclein and post-translationally modified species (i.e., phosphorylated α-synuclein) as early indicators before typical Lewy pathology may be detected in PD and related diseases. This phenomenon can, therefore, be seen as a prodromal pathological feature to PD.

## Conclusion

In conclusion, for the first time, our study shows that α-synuclein and phosphorylated α-synuclein accumulation in the cerebral motor cortex, pre-commissural putamen and dopaminergic neurons of the substantia nigra of aged cynomolgus monkeys with spontaneous T2DM compared to the age-matched normal subjects. We also observed correlative neuropathological alterations of PD with the severity of diabetes and dyslipidemia. These findings strongly implicated the close association of pathogeneses between diabetes and PD. Further studies are warranted to verify the underlying molecular mechanisms between T2DM and PD.

## Data Availability Statement

All dataset generated for this study are included in the article/Supplementary Material.

## Ethics Statement

All the procedures were reviewed and approved by the Institutional Animal Care and Use Committee (IACUC) of Wincon TheraCells Biotechnologies Co., Ltd. (Wincon) in Nanning, Guangxi, China, which is fully accredited by the Association for Assessment and Accreditation of Laboratory Animal Care (AAALAC).

## Author Contributions

J-YL and FY conceived and coordinated the study. YS and J-YL designed the experiments and wrote the manuscript. YS performed the experiments. YS, CG, LY, WL, and J-YL analyzed and interpreted the data. Z-YW and FY provide the intellectual input. All authors reviewed and contributed to the writing, read and approved the final manuscript.

## Conflict of Interest

The authors declare that the research was conducted in the absence of any commercial or financial relationships that could be construed as a potential conflict of interest.

## Abbreviations

A β: amyloid- β
AD: Alzheimer’s disease
CHOL: cholesterol
DA: dopaminergic
DLB: Dementia with Lewy Body
DM: diabetes mellitus
FPG: fasting plasma glucose
HDL: high density lipoprotein
IAPP: islet amyloid polypeptide
LBs: Lewy Bodies
LDL: low density lipoprotein
LNs: Lewy neurites
MSA: multiple system atrophy
PCC: Pearson correlation coefficient
PD: Parkinson’s disease
PFA: paraformaldehyde
PNS: peripheral nervous system
PrP: prion protein
SNpc: substantia nigra pars compacta
T1DM: Type 1 Diabetes Mellitus
T2DM: Type 2 Diabetes Mellitus
TG: triglyceride
TH: tyrosine hydroxylase.
