# Supplementary material for: Cynomolgus Monkeys With Spontaneous Type-2-Diabetes-Mellitus-Like Pathology Develop Alpha-Synuclein Alterations Reminiscent of Prodromal Parkinson’s Disease and Related Diseases
Source: Front Neurosci. 2020 Feb 7;14:63. doi: 10.3389/fnins.2020.00063 (PMC7019001; doi:10.3389/fnins.2020.00063)
Supplement: Supplementary file 3 [file Presentation_1.PPTX]

## Slide 1
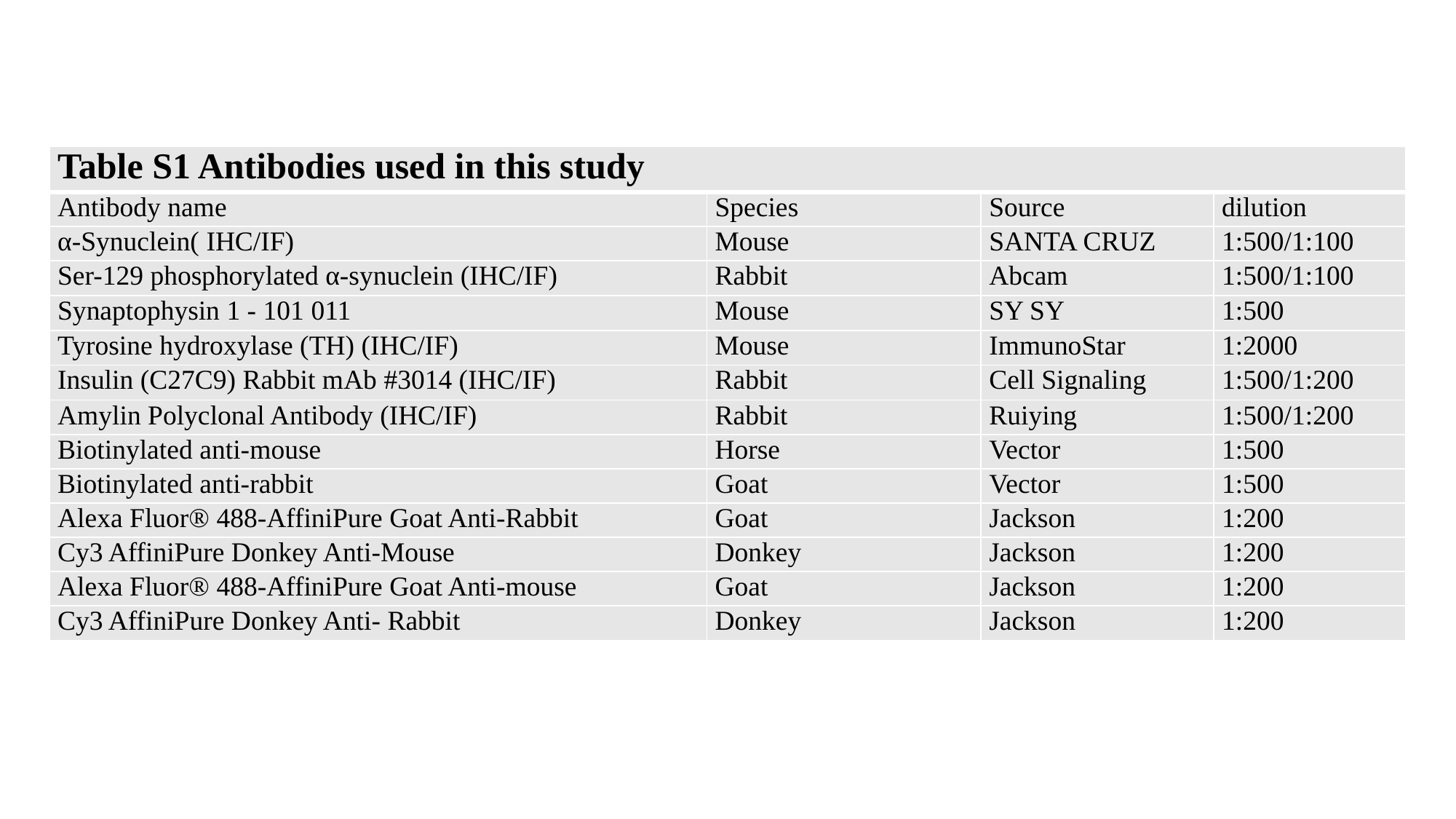

| Table S1 Antibodies used in this study | | | |
| --- | --- | --- | --- |
| Antibody name | Species | Source | dilution |
| α-Synuclein( IHC/IF) | Mouse | SANTA CRUZ | 1:500/1:100 |
| Ser-129 phosphorylated α-synuclein (IHC/IF) | Rabbit | Abcam | 1:500/1:100 |
| Synaptophysin 1 - 101 011 | Mouse | SY SY | 1:500 |
| Tyrosine hydroxylase (TH) (IHC/IF) | Mouse | ImmunoStar | 1:2000 |
| Insulin (C27C9) Rabbit mAb #3014 (IHC/IF) | Rabbit | Cell Signaling | 1:500/1:200 |
| Amylin Polyclonal Antibody (IHC/IF) | Rabbit | Ruiying | 1:500/1:200 |
| Biotinylated anti-mouse | Horse | Vector | 1:500 |
| Biotinylated anti-rabbit | Goat | Vector | 1:500 |
| Alexa Fluor® 488-AffiniPure Goat Anti-Rabbit | Goat | Jackson | 1:200 |
| Cy3 AffiniPure Donkey Anti-Mouse | Donkey | Jackson | 1:200 |
| Alexa Fluor® 488-AffiniPure Goat Anti-mouse | Goat | Jackson | 1:200 |
| Cy3 AffiniPure Donkey Anti- Rabbit | Donkey | Jackson | 1:200 |

## Slide 2
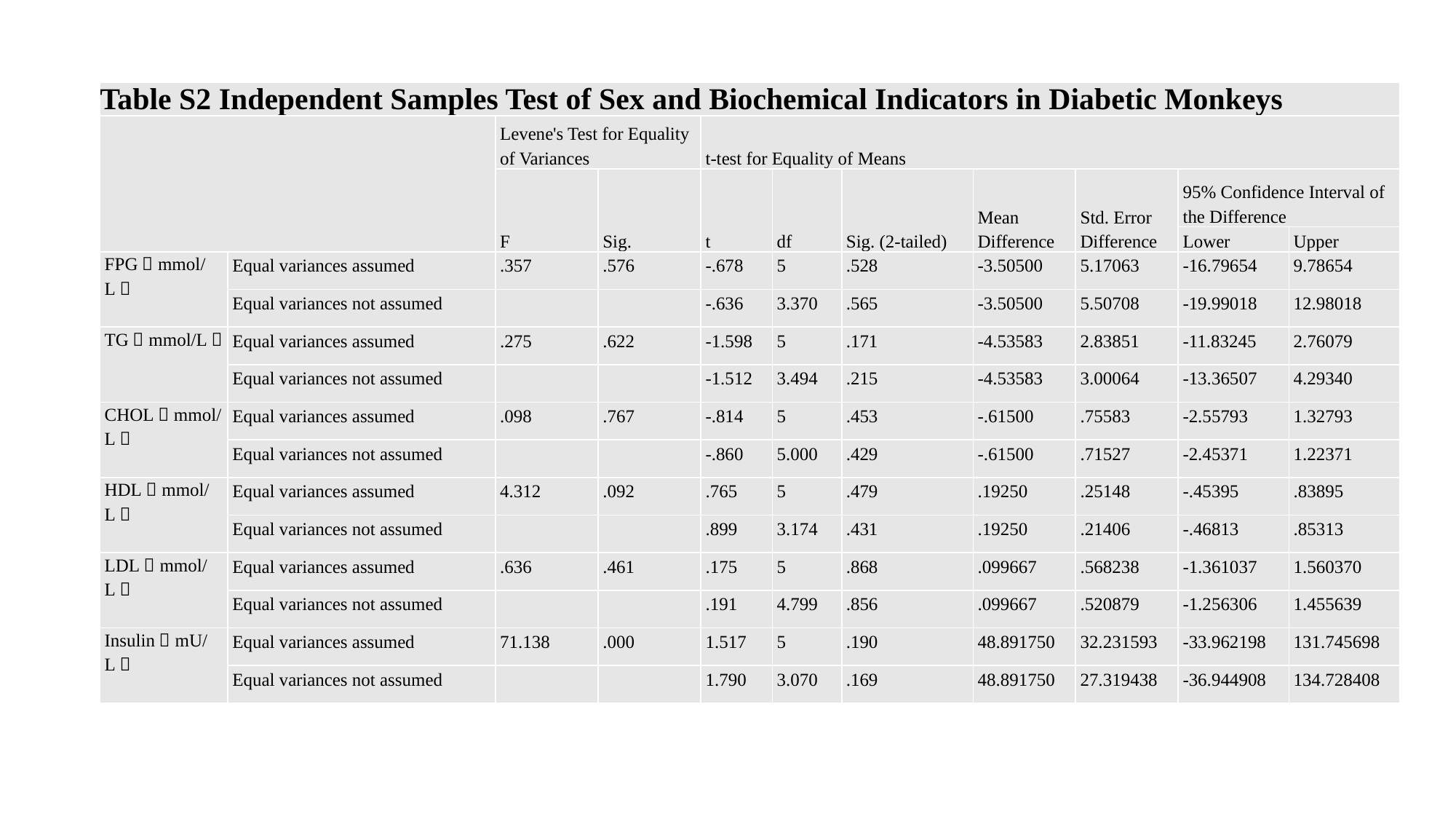

| Table S2 Independent Samples Test of Sex and Biochemical Indicators in Diabetic Monkeys | | | | | | | | | | |
| --- | --- | --- | --- | --- | --- | --- | --- | --- | --- | --- |
| | | Levene's Test for Equality of Variances | | t-test for Equality of Means | | | | | | |
| | | F | Sig. | t | df | Sig. (2-tailed) | Mean Difference | Std. Error Difference | 95% Confidence Interval of the Difference | |
| | | | | | | | | | Lower | Upper |
| FPG（mmol/L） | Equal variances assumed | .357 | .576 | -.678 | 5 | .528 | -3.50500 | 5.17063 | -16.79654 | 9.78654 |
| | Equal variances not assumed | | | -.636 | 3.370 | .565 | -3.50500 | 5.50708 | -19.99018 | 12.98018 |
| TG（mmol/L） | Equal variances assumed | .275 | .622 | -1.598 | 5 | .171 | -4.53583 | 2.83851 | -11.83245 | 2.76079 |
| | Equal variances not assumed | | | -1.512 | 3.494 | .215 | -4.53583 | 3.00064 | -13.36507 | 4.29340 |
| CHOL（mmol/L） | Equal variances assumed | .098 | .767 | -.814 | 5 | .453 | -.61500 | .75583 | -2.55793 | 1.32793 |
| | Equal variances not assumed | | | -.860 | 5.000 | .429 | -.61500 | .71527 | -2.45371 | 1.22371 |
| HDL（mmol/L） | Equal variances assumed | 4.312 | .092 | .765 | 5 | .479 | .19250 | .25148 | -.45395 | .83895 |
| | Equal variances not assumed | | | .899 | 3.174 | .431 | .19250 | .21406 | -.46813 | .85313 |
| LDL（mmol/L） | Equal variances assumed | .636 | .461 | .175 | 5 | .868 | .099667 | .568238 | -1.361037 | 1.560370 |
| | Equal variances not assumed | | | .191 | 4.799 | .856 | .099667 | .520879 | -1.256306 | 1.455639 |
| Insulin（mU/L） | Equal variances assumed | 71.138 | .000 | 1.517 | 5 | .190 | 48.891750 | 32.231593 | -33.962198 | 131.745698 |
| | Equal variances not assumed | | | 1.790 | 3.070 | .169 | 48.891750 | 27.319438 | -36.944908 | 134.728408 |

## Slide 3
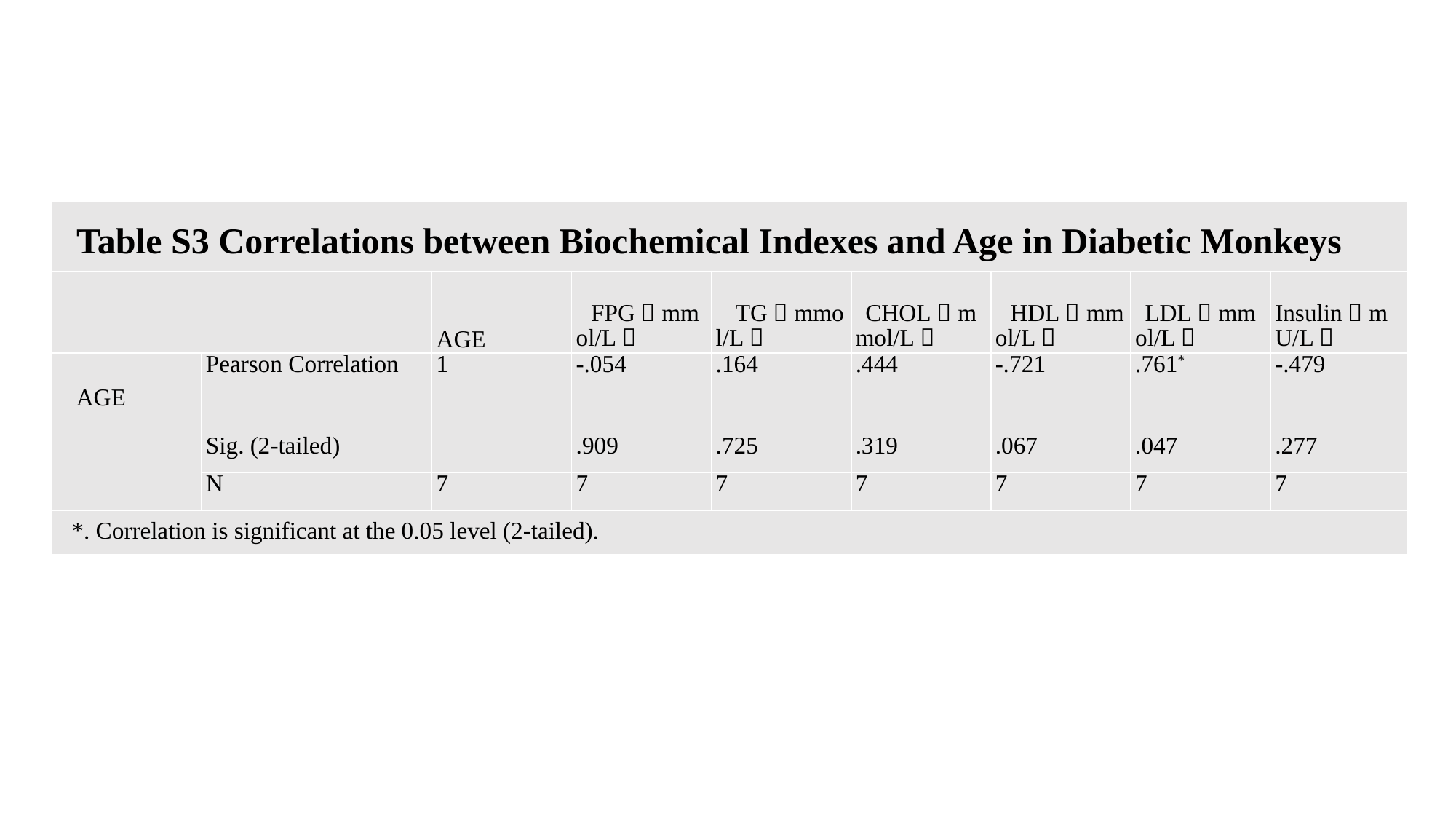

| Table S3 Correlations between Biochemical Indexes and Age in Diabetic Monkeys | | | | | | | | |
| --- | --- | --- | --- | --- | --- | --- | --- | --- |
| | | AGE | FPG（mmol/L） | TG（mmol/L） | CHOL（mmol/L） | HDL（mmol/L） | LDL（mmol/L） | Insulin（mU/L） |
| AGE | Pearson Correlation | 1 | -.054 | .164 | .444 | -.721 | .761\* | -.479 |
| | Sig. (2-tailed) | | .909 | .725 | .319 | .067 | .047 | .277 |
| | N | 7 | 7 | 7 | 7 | 7 | 7 | 7 |
| \*. Correlation is significant at the 0.05 level (2-tailed). | | | | | | | | |

## Slide 4
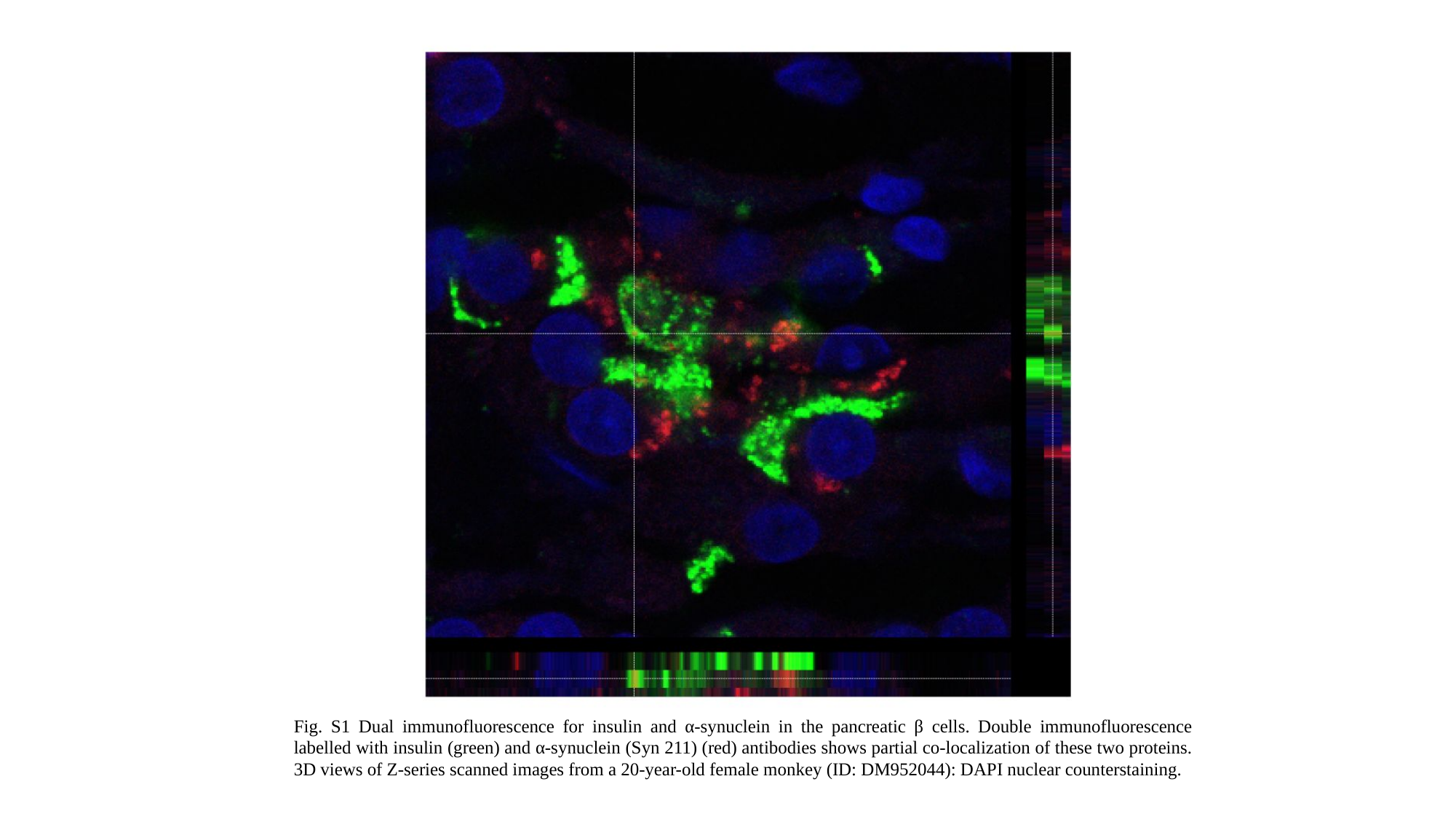

Fig. S1 Dual immunofluorescence for insulin and α-synuclein in the pancreatic β cells. Double immunofluorescence labelled with insulin (green) and α-synuclein (Syn 211) (red) antibodies shows partial co-localization of these two proteins. 3D views of Z-series scanned images from a 20-year-old female monkey (ID: DM952044): DAPI nuclear counterstaining.

## Slide 5
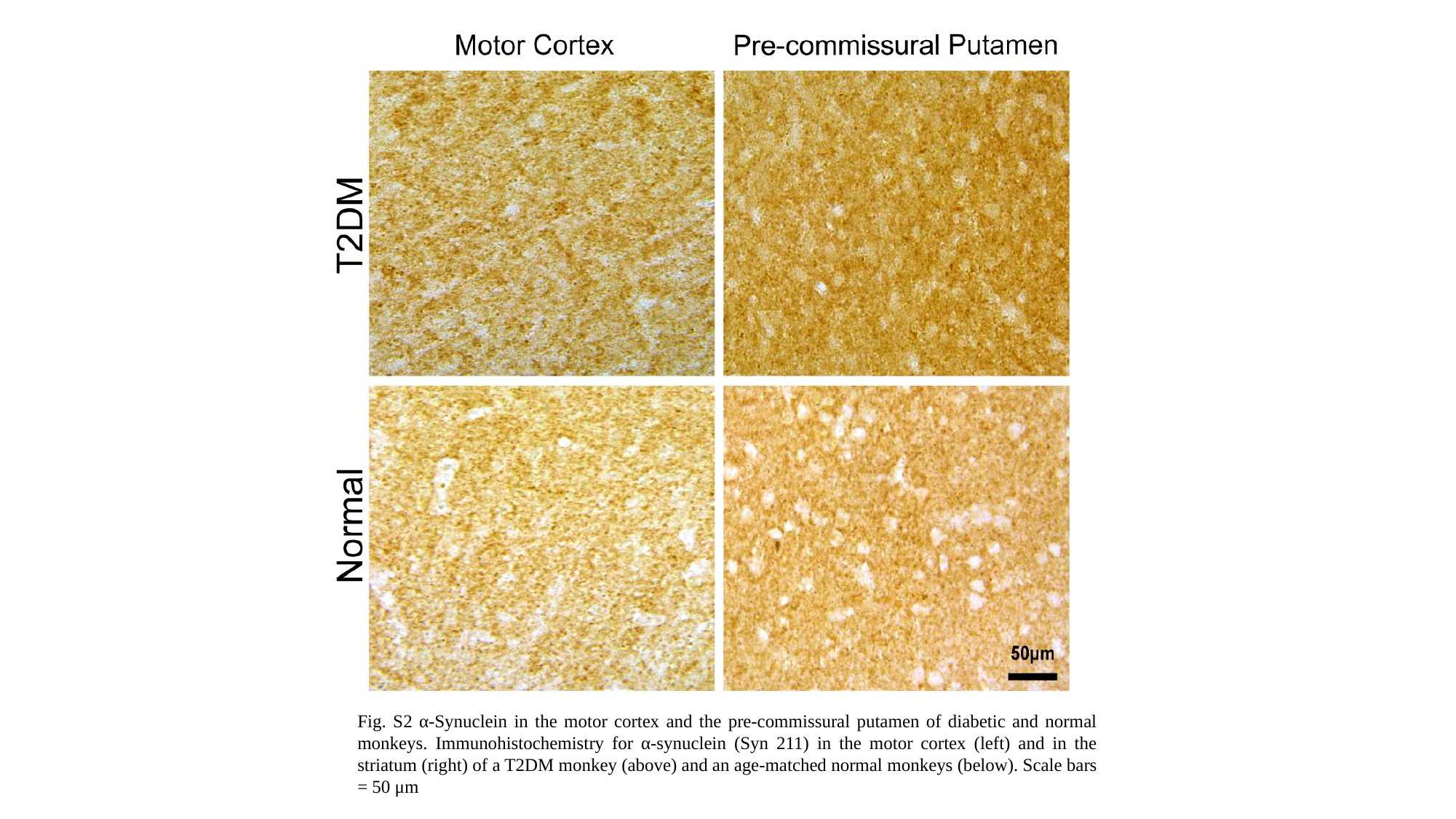

Fig. S2 α-Synuclein in the motor cortex and the pre-commissural putamen of diabetic and normal monkeys. Immunohistochemistry for α-synuclein (Syn 211) in the motor cortex (left) and in the striatum (right) of a T2DM monkey (above) and an age-matched normal monkeys (below). Scale bars = 50 μm

## Slide 6
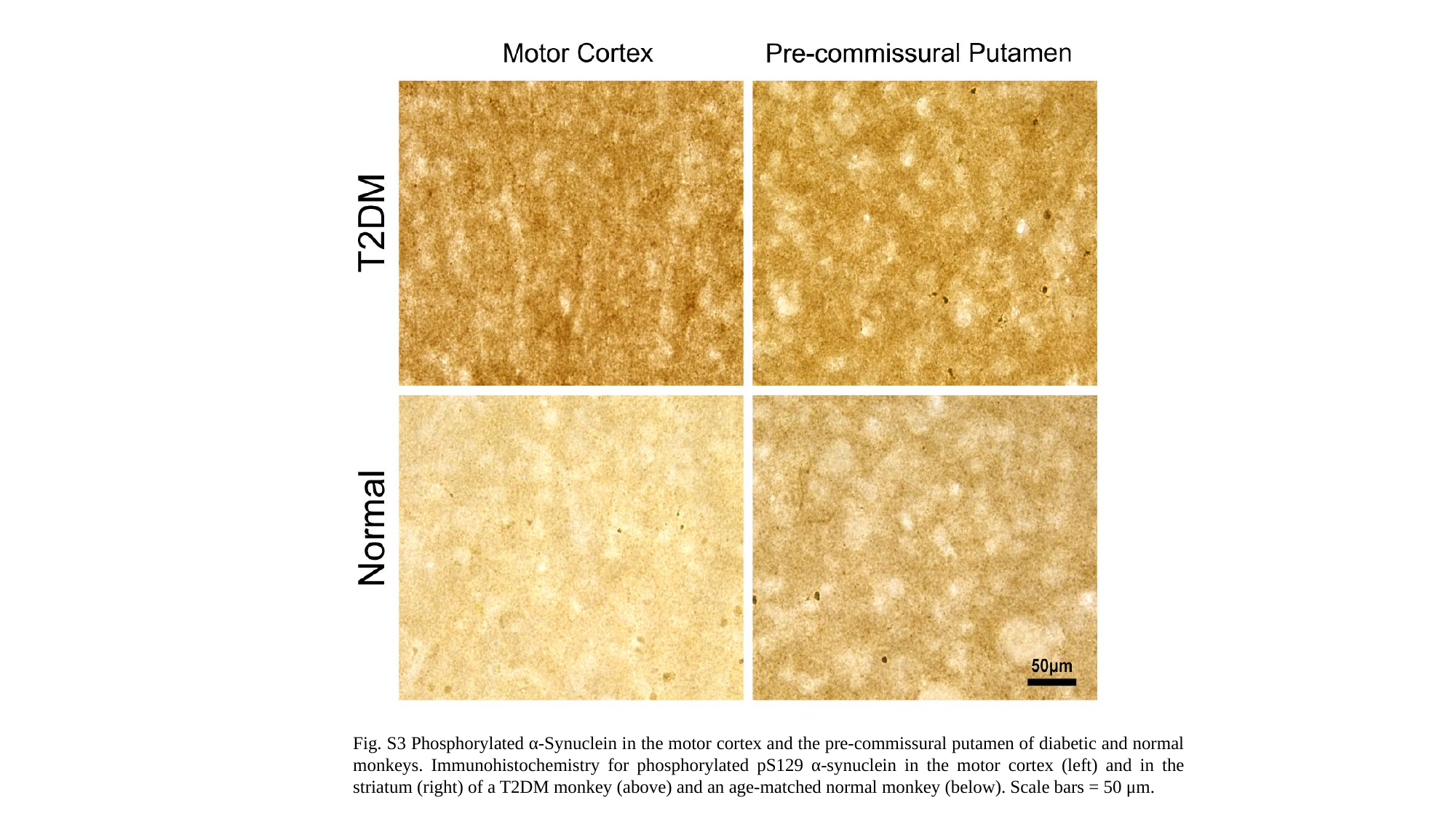

Fig. S3 Phosphorylated α-Synuclein in the motor cortex and the pre-commissural putamen of diabetic and normal monkeys. Immunohistochemistry for phosphorylated pS129 α-synuclein in the motor cortex (left) and in the striatum (right) of a T2DM monkey (above) and an age-matched normal monkey (below). Scale bars = 50 μm.

## Slide 7
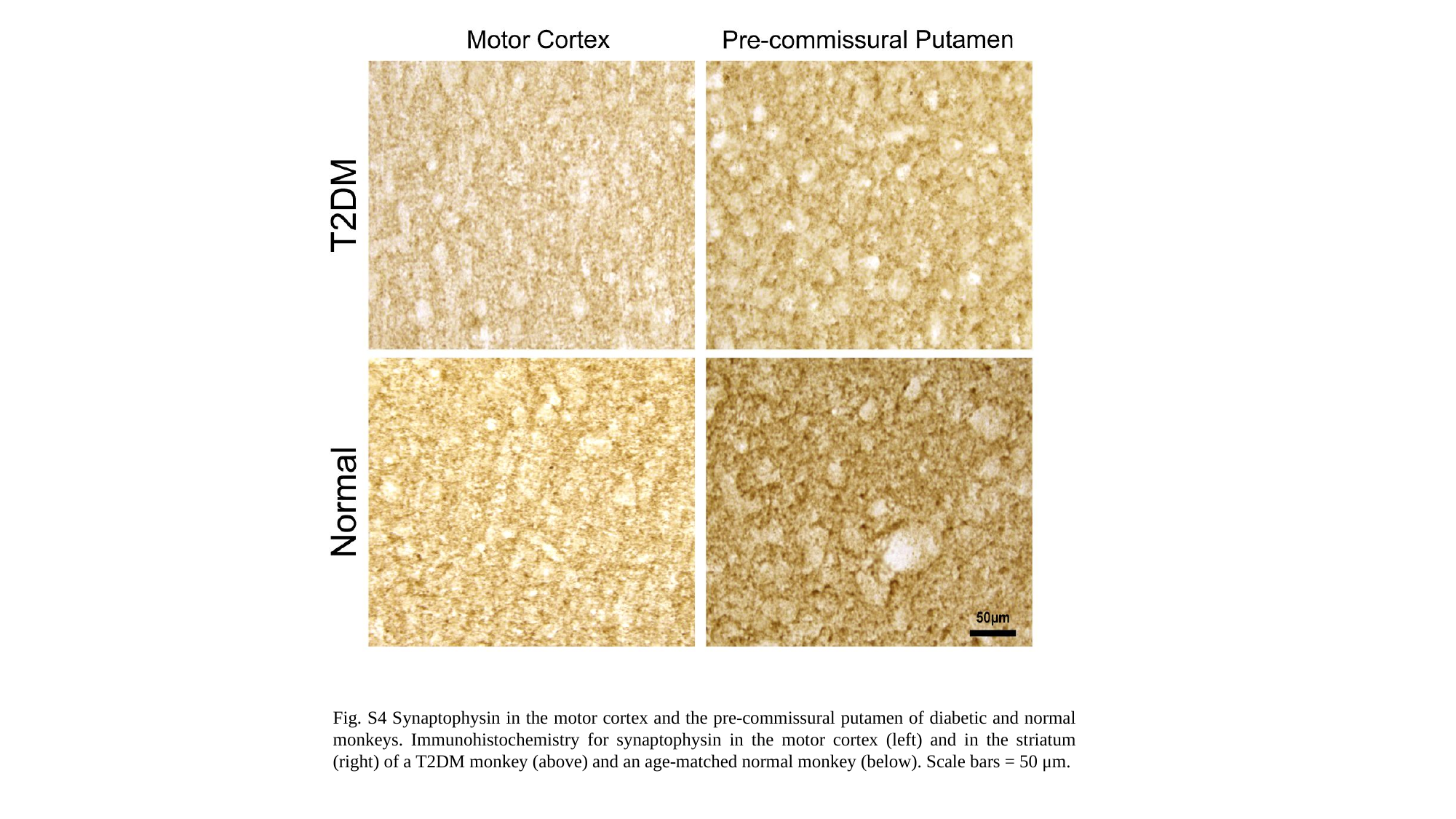

Fig. S4 Synaptophysin in the motor cortex and the pre-commissural putamen of diabetic and normal monkeys. Immunohistochemistry for synaptophysin in the motor cortex (left) and in the striatum (right) of a T2DM monkey (above) and an age-matched normal monkey (below). Scale bars = 50 μm.

## Slide 8
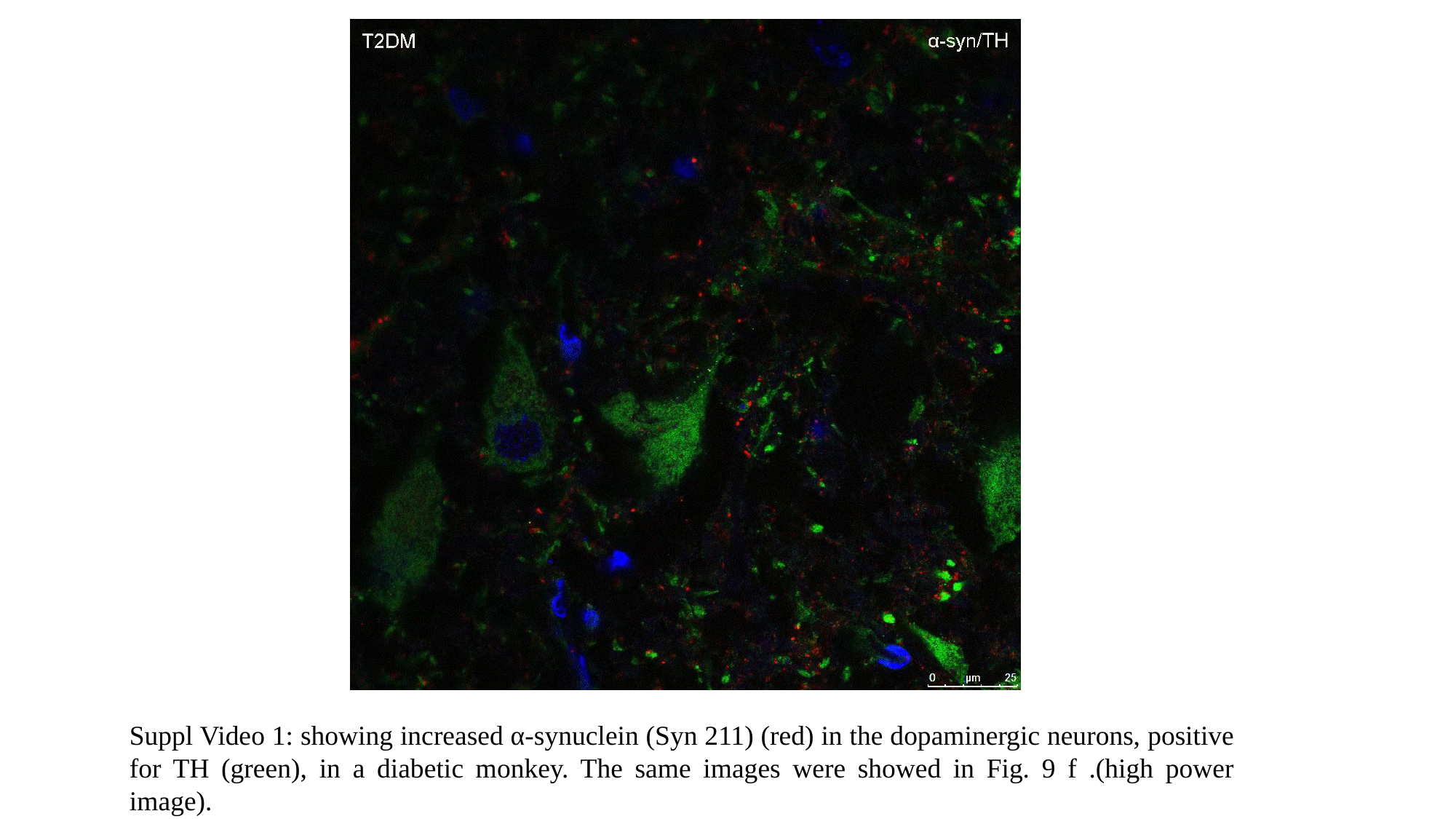

Suppl Video 1: showing increased α-synuclein (Syn 211) (red) in the dopaminergic neurons, positive for TH (green), in a diabetic monkey. The same images were showed in Fig. 9 f .(high power image).

## Slide 9
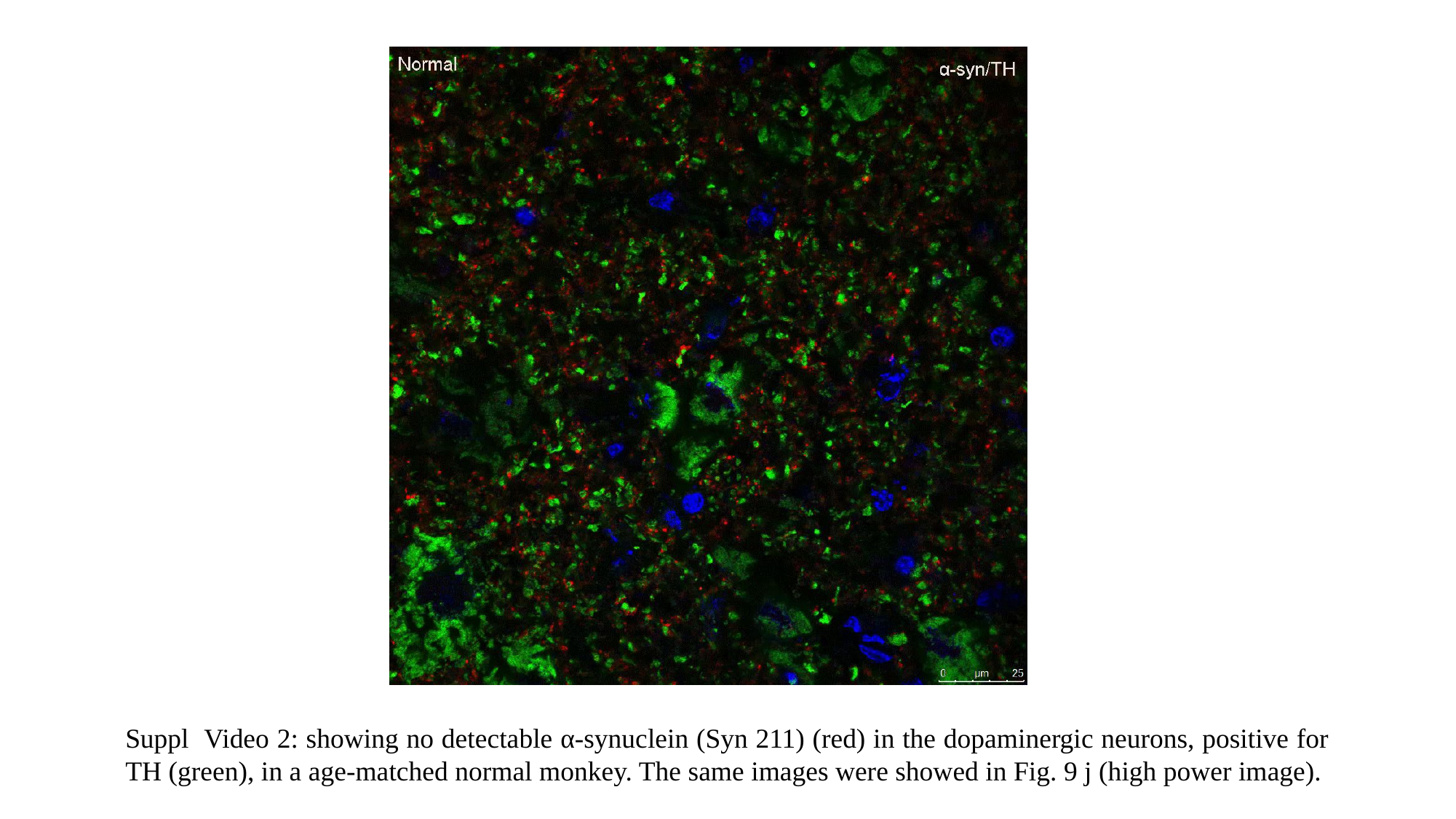

Suppl Video 2: showing no detectable α-synuclein (Syn 211) (red) in the dopaminergic neurons, positive for TH (green), in a age-matched normal monkey. The same images were showed in Fig. 9 j (high power image).
